# Association between metabolic syndrome and knee osteoarthritis: a cross-sectional study

**DOI:** 10.1186/s12891-017-1890-9

**Published:** 2017-12-16

**Authors:** Dong-xing Xie, Jie Wei, Chao Zeng, Tuo Yang, Hui Li, Yi-lun Wang, Hui-zhong Long, Zi-ying Wu, Yu-xuan Qian, Kang-hua Li, Guang-hua Lei

**Affiliations:** 1 Department of Orthopaedics, Xiangya Hospital, Central South University, #87 Xiangya Road, Changsha, Hunan 410008 People’s Republic of China; 2 Health Management Center, Xiangya Hospital, Central South University, Changsha, Hunan Province 410008 People’s Republic of China; 3 Department of Epidemiology and Health Statistics, Xiangya School of Public Health, Central South University, Changsha, Hunan Province 410008 People’s Republic of China

**Keywords:** Osteoarthritis, Metabolic syndrome, Knee

## Abstract

**Background:**

Osteoarthritis (OA) is the most prevalent chronic joint disease in China. The aim of this study was to examine the association between metabolic syndrome (MetS) and knee OA in a population-based Chinese study.

**Methods:**

Data included in this analysis is from a cross-sectional study, i.e., the Xiangya Hospital Health Management Center Study. MetS was diagnosed according to the criteria defined by the Chinese Diabetes Society. Radiographic knee OA was defined as changes equivalent to Kellgren-Lawrence (K-L) grade 2 or above at least one side. Associations between MetS and its components with OA were evaluated by conducting multivariable adjusted logistic regression.

**Results:**

A total of 5764 participants were included in the present study. The unadjusted OR (1.27, 95%CI: 1.10–1.47, *P* = 0.001), age-sex adjusted OR (1.17, 95%CI: 1.01–1.36, *P* = 0.041) and multivariable adjusted OR (1.17, 95%CI: 1.01–1.36, *P* = 0.043) all suggested a positive association between MetS and knee OA. Besides, its components (e.g., overweight, hypertension and dyslipidemia) were also associated with the prevalence of radiographic knee OA respectively, after adjusting for some confounding factors. In addition, with the accumulation of MetS components, the prevalence of knee OA increased. Furthermore, MetS as a whole was associated with the prevalence of knee osteophyte (OSP) (OR = 1.72, 95%CI: 1.42–2.09, *P* < 0.001), but not joint space narrowing (JSN) (OR = 1.06, 95%CI: 0.91–1.23, *P* = 0.449).

**Conclusions:**

The findings of the present study indicated that there was a positive association between the prevalence of MetS and knee OA. However, MetS as a whole was associated with the higher prevalence of knee OSP, but not JSN, which should shed light on our understanding the association between MetS and OA.

## Background

Osteoarthritis (OA) is the most prevalent chronic joint disease and a major cause of pain and disability worldwide [1]. Although the pathophysiologic mechanisms of OA are inconclusive, growing evidence has supported that metabolic factors may contribute to the initiation and progression of OA process [2]. Epidemiological studies have demonstrated a positive association between OA and several metabolic risk factors, such as dyslipidemia, hyperglycaemia and hypertension [3–5]. Metabolic syndrome (MetS) is a common metabolic disorder that results from the increasing prevalence of obesity and associated with an increased risk of cardiovascular disease [2, 6]. Recently, metabolic OA has been nominated as the fifth component of MetS [2], since OA was classified into three phenotypes including metabolic OA, age-related OA and injure-related OA [7]. In view of the shared mechanisms, scholars concluded that MetS is closely related to OA, and OA is even a part of the generalized metabolic disorder [2, 7–9].

OA is characterized by the pathologic features of joint space narrowing (JSN) and osteophyte (OSP) formation. Because accumulating evidences have shown that these two abnormalities have distinct etiologic mechanism [10–12], it would be helpful to elucidate the pathogenesis of MetS or OA by gaining more in-depth understanding of the associations of MetS with JSN and OSP. During the past several decades, the Chinese population has experienced remarkable changes in lifestyle as economic development and the ageing process went on [13]. As a consequence, a variety of health problems emerged, such as the increase of the prevalence of MetS and OA [14–18]. Recently, a case-control study which included 70 end-stage OA patients and a matched control group conducted in China revealed a correlation between MetS and OA [19]. However, due to the small sample size and only grade IV OA being involved in this study, the possible applicability of the results to the general Chinese population, as the authors acknowledged, would be limited. To our best knowledge, no analysis based on a large sample has examined the association between MetS and OA regarding general Chinese population.

To bridge the knowledge gap, we used data collected from a large population-based study (i.e., *Xiangya Hospital Health Management Center Study*) and examined the relation of MetS and its components with the prevalence of radiographic knee OA and to explore the association between MetS with OSP and JSN respectively.

## Methods

### Study population of cross-sectional study

This observational study was conducted in the Department of Health Examination Center Xiangya Hospital, Central South University in Changsha, Hunan Province, China. It was approved by the ethics committee at Xiangya Hospital, Central South University. Also, written informed consent was obtained from the participants in our study. The study design has been published in peer-reviewed journals [20–22]. Besides, some part of the detailed methodology, including study population, assessment of radiographic knee OA, blood biochemical analyses and assessment of other exposures, in the present study were also described in our previously published work [23–25]. In order to collect information on demographic characteristics and health-related habits, a standardized questionnaire was used by registered nurses to interview all participants during the examination. As previously described [24], participants were selected according to the following inclusion criteria: 1) ≥ 40 years old; 2) undergone weight-bearing bilateral anteroposterior radiography of the knee; 3) availability of all basic characteristics, including age, gender, body mass index (BMI), blood pressure etc.; 4) availability of biochemical test results; 5) availability of information related to the living habits, including education background, activity level, smoking, drinking and medication status. At the beginning, 13,631 subjects were undergone routine checkups including knee radiography at the Department of Health Examination Center Xiangya Hospital, Central South University in Changsha, Hunan Province, China, from October 2013 to November 2014. Finally, 5764 participants were included in the present study (132 diagnosed with other joint diseases based on radiographic evidence, such as osteochondroma or other bone tumors; 191 participants were not available of biochemical test results; 440 participants were not available of basic characteristics; 1207 participants were younger than 40 years old; 5897 did not offer the information of living habits).

### Assessment of radiographic knee OA

As described in our previous work [21], all subjects included in the present cross-sectional study had undergone weight-bearing bilateral anteroposterior radiography of the knee. Two orthopedists, blinded to subjects’ basic information, assessed the radiographs independently by using the Kellgren-Lawrence (K-L) radiographic atlas. Inconsistent opinions were resolved through discussions. The weighted kappa coefficient for knee x-Ray scoring was 0.85 for inter-reader reliability and 0.86 for intra-reader reliability. The severity of OA was classified into five grades according to the K-L atlas: 0 = absence of OA; 1 = suspected OA; 2 = minimal OA; 3 = moderate OA; 4 = severe OA [26]. A participant would be considered as radiographic knee OA if at least one of his/her knee joint was graded K-L 2 or above, however, pain was not taken into account and symptomatic knee OA was not diagnosed in the present study. In addition, JSN and OSP were assessed individually based on a scale of 0–3 (0 = normal; 3 = most severe) according to the Osteoarthritis Research Society International (OARSI) atlas [27].

### Assessment of MetS

MetS was diagnosed according to the Chinese Diabetes Society (CDS) criteria [28–30]. CDS criteria for MetS requires 3 items or all of the four items: (1) BMI ≥ 25 kg/m^2^; (2) Fasting plasma glucose (FPG) ≥ 6.1 mmol/L, or treatment of diagnosed diabetes; (3) Systolic blood pressure (BP) ≥ 140 mmHg or diastolic BP ≥ 90 mmHg, or treatment of previously diagnosed hypertension; (4) Triglycerides ≥1.7 mmol/L and/or HDL-cholesterol <0.9 mmol/L in male or <1.0 mmol/L in female, or treatment for this lipid abnormality. Besides, the NCEP-ATPIII (Asian revised version) criteria [31, 32] was also used to assess MetS and it was based on presence of at least three of following risk factors: waist circumference ≥ 90 cm in men and ≥80 cm in women; triglycerides ≥150 mg/dL (1.7 mmol/L); HDL cholesterol <40 mg/dL (1.03 mmol/L) in men and <50 mg/dL (1.29 mmol/L) in women; systolic BP ≥ 130 mmHg, diastolic BP ≥ 85 mmHg, or currently using antihypertensive medication; and FPG ≥ 100 mg/dL (5.6 mmol/L) or currently undergone drug treatment for blood glucose control.

### Blood biochemistry

All blood samples were drawn after a 12-h overnight fast and were kept at 4 °C until analysis. The fasting blood glucose concentration and blood lipid assessment were performed using a Beckman Coulter AU 5800 (Beckman Coulter Inc., Brea, CA, USA). The high sensitivity C reactive protein (hsCRP) was measured by Latex turbidity method. The inter- and intra-assay coefficients of variation were tested by low concentrations (2.5 mmol/L for glucose) and high concentrations (6.7 mmol/L for glucose) of standard human samples. The intra-assay coefficients of variation were 0.98% (2.5 mmol/L) and 1.72% (6.7 mmol/L) for glucose. The inter-assay coefficients of variation were 2.45% (2.5 mmol/L) and 1.46% (6.7 mmol/L) for glucose.

### Assessment of other exposures

Assessment of other exposures was also described in our previous work [21]. Specifically, blood pressure of each participant was measured using an electronic sphygmomanometer. The weight and height of each participant was measured respectively to calculate the BMI. Participants were also asked about their average frequency of physical activity (never, one to two times per week, three to four times per week, five times and above per week) and average duration of physical activity (within half an hour, half an hour to one hour, one to two hours, more than two hours) through a standard questionaire. The smoking and alcohol drinking status were asked face to face.

### Statistical analysis

The continuous data are expressed as mean ± standard deviation, and the category data are expressed in percentage. Differences in continuous data were evaluated by Mann-Whitney U test, while differences in category data were assessed by the χ^2^ test.

Associations between knee OA with MetS and MetS components in the present cross-sectional study were evaluated by conducting age-sex adjusted and multivariable adjusted logistic regression. The following confounding factors were considered to be included in the multivariable model: age, sex, activity level, smoking status, alcohol drinking status and educational background [33–35]. Sensitivity analysis was conducted through adding hsCRP into the multivariable model. Associations between MetS with JSN and OSP were also assessed by multivariable adjusted logistic regression. We also assessed the association between OA and the number of MetS components through multivariable adjusted logistic regression. Moreover, the association between MetS diagnosed according to the NCEP-ATPIII (Asian revised version) criteria was examined. Crude OR, age-sex-BMI adjusted OR and multivariable (BMI, age, sex, activity level, smoking status, alcohol drinking status and educational background) adjusted OR and their related 95%CI were calculated for evaluating the associations. Besides, the association between MetS and radiographic severity of knee OA was also examined.

## Results

As previously described [23], a total of 5764 subjects were included in the present cross-sectional study. The characteristics of the study population in terms of knee OA status were illustrated in Table 1. The overall prevalence of OA and MetS in the target population was 29.0% and 17.7%, respectively. The prevalence of MetS in knee OA subjects (20.3%) was significantly higher than non-knee OA subjects (16.6%) (*P* = 0.001). Significant differences were observed between knee OA and non-knee OA subjects in terms of the age, sex, BMI, fasting glucose, blood pressure, HDL-cholesterol, and triglyceride.

**Table 1:** Basic characteristics of included subjects according to OA status (*n* = 5764)

	OA subjects	Non-OA subjects	*P*
Participants (n)	1669	4095	–
MetS (%)	20.3	16.6	0.001
Age (years)	55.84 (8.01)	51.81 (6.84)	<0.001
BMI (kg/m^2^)	24.84 (3.36)	24.36 (3.14)	<0.001
Female (%)	43.3	46.8	0.018
Smoking (%)	21.2	22.5	0.251
Alcohol drinking (%)	37.1	38.9	0.223
High school diploma (%)	46.2	47.7	0.306
Activity level (h/w)	2.49 (3.71)	2.20 (3.43)	0.069
Fasting glucose (mmol/l)	5.81 (1.74)	5.66 (1.62)	<0.001
systolic pressure (mm Hg)	128.56 (17.20)	125.40 (17.49)	<0.001
Diastolic pressure (mm Hg)	80.62 (11.39)	79.92 (12.13)	0.008
HDL-cholesterol (mmol/l)	1.49 (0.38)	1.52 (0.40)	0.041
Triglyceride (mmol/l)	1.96 (1.85)	1.93 (1.81)	0.476
HsCRP (mg/l)	2.30 (4.95)	2.39 (5.56)	0.755

OA, osteoarthritis; MetS, metabolic syndrome; BMI, body mass index; HDL, high-density lipoprotein; hsCRP, high sensitivity C reactive protein

Outcomes of unadjusted, age-sex adjusted and multivariable adjusted associations (age, sex, activity level, smoking status, alcohol drinking status and educational background) between MetS and knee OA were shown in Table 2. The unadjusted OR (1.27, 95%CI: 1.10–1.47, *P* = 0.001), age-sex adjusted OR (1.17, 95%CI: 1.01–1.36, *P* = 0.041) and multivariable adjusted OR (1.17, 95%CI: 1.01–1.36, *P* = 0.043) all suggested a positive association between MetS and OA. In addition, all the MetS components except for hyperglycaemia in the age-sex adjusted and multivariable adjusted models were associated with knee OA. In the two aforementioned models, the association between hyperglycaemia and knee OA approached significant. The relationships between the accumulation of MetS components and the prevalence of radiographic knee OA were presented in Table 3. Generally, the prevalence of knee OA increased with the accumulation of MetS components. The multivariable adjusted ORs are as follows: one component, 1.36, 95%CI: 1.14–1.61, P = 0.001; two components, 1.99, 95%CI: 1.67–2.38, *P* < 0.001 and three or more components, 2.12, 95%CI: 1.74–2.57, *P* < 0.001.

**Table 2:** Associations between MetS and OA in the present cross-sectional study (*n* = 5764)

				Model 1^a^			Model 2^a^		
	Unadjusted OR	95%CI	*P*	Adjusted OR	95%CI	*P*	Adjusted OR	95%CI	*p*
MetS as a whole	1.27	1.10–1.47	0.001	1.17	1.01–1.36	0.041	1.17	1.01–1.36	0.043
MetS components									
Overweight	2.85	2.54–3.21	<0.001	2.14	1.89–2.43	<0.001	2.15	1.90–2.44	<0.001
Hyperglycaemia	1.36	1.18–1.57	<0.001	1.15	0.99–1.33	0.067	1.15	0.99–1.33	0.066
Hypertension	1.45	1.29–1.63	<0.001	1.23	1.09–1.40	0.001	1.23	1.09–1.40	0.001
Dyslipidemia	1.53	1.37–1.72	<0.001	1.33	1.18–1.50	<0.001	1.33	1.18–1.50	<0.001

MetS, metabolic syndrome; OA, osteoarthritis;

^a^Model 1 was adjusted for age and sex; Model 2 was adjusted for age, sex, activity level, smoking status, alcohol drinking status and educational background

**Table 3:** Associations between number of MetS components and OA in the present cross-sectional study

	N (%)	OA (%)	Adjusted OR	95%CI	*p*
Without MetS component	1640 (28.5)	292 (17.8)	1.00	reference	–
With one MetS component	1709 (29.6)	446 (26.1)	1.36	1.14–1.61	0.001
With two MetS components	1396 (24.2)	508 (36.4)	1.99	1.67–2.38	<0.001
With three or four MetS components	1019 (17.7)	423 (41.5)	2.12	1.74–2.57	<0.001

MetS, metabolic syndrome; OA, osteoarthritis;

Multivariable model was was adjusted for age, sex, activity level, smoking status, alcohol drinking status and educational background

The result of sensitivity analysis shown that the multivariable adjusted association between MetS and knee OA remained significant after adding hsCRP into the multivariable model (OR, 1.45, 95%CI: 1.12–1.87, *P* = 0.004). The associations between MetS and its components with JSN and OSP were shown in Table 4. Only MetS as a whole was associated with knee OSP (OR = 1.72, 95%CI: 1.42–2.09, *P* < 0.001), but not JSN (OR = 1.06, 95%CI: 0.91–1.23, *P* = 0.449). In addition, overweight (OR = 1.26, 95%CI: 1.11–1.43, *P* < 0.001) and dyslipidemia (OR = 1.18, 95%CI: 1.05–1.33, *P* = 0.005) were associated with JSN; overweight (OR = 1.61, 95%CI: 1.36–1.90, *P* < 0.001) and hypertension (OR = 1.24, 95%CI: 1.06–1.46, *P* = 0.009) were associated with OSP. It should be noted that the positive association between MetS and radiographic knee OA also existed (crude OR = 1.29, 1.14–1.49, *P* < 0.001; age-sex-BMI adjusted OR = 1.14, 95%CI: 1.10–1.29, *P* = 0.058; multivariable adjusted OR = 1.14, 95%CI: 1.00–1.30, *P* = 0.052) when MetS was diagnosed based on the NCEP-ATPIII (Asian revised version) criteria. Besides, MetS was also associated with radiographic severity of knee OA (multivariable adjusted OR = 1.12, 95%CI: 0.99–1.27, *P* = 0.08).

**Table 4:** Associations between MetS and its components with JSN and OSP in the present cross-sectional study (*n* = 5764)

	MetS	Overweight	Hyperglycaemia	Hypertension	Dyslipidemia
JSN					
Adjusted OR	1.06	1.26	1.10	1.03	1.18
95%CI	0.91–1.23	1.11–1.43	0.95–1.27	0.91–1.17	1.05–1.33
P	0.449	<0.001	0.197	0.626	0.005
OSP					
Adjusted OR	1.72	1.61	1.16	1.24	1.16
95%CI	1.42–2.09	1.36–1.90	0.96–1.40	1.06–1.46	0.99–1.36
P	<0.001	<0.001	0.132	0.009	0.065

MetS, metabolic syndrome; OA, osteoarthritis; JSN, joint space narrowing; OSP, osteophyte;

Multivariable model was adjusted for age, sex, activity level, smoking status, alcohol drinking status and educational background

## Discussion

Based on a relatively large-scale population-based cross-sectional study, it was found that MetS and its components (e.g., overweight, hypertension and dyslipidemia) were associated with the prevalence of radiographic knee OA in a Chinese population with adjustment of a number of confounding factors. With the accumulation of MetS components, the prevalence of knee OA increased. The positive association remained significant after adding hsCRP into the multivariable model. In addition, MetS as a whole was only associated with knee OSP, but not JSN.

Notably, the present study found that MetS as a whole was associated with knee OSP, but not JSN, which is consistent with a previous cross-sectional study. In that study, Yoshimura et al. illustrated that the number of MetS components (e.g., overweight, hypertension, dyslipidemia, and impaired glucose tolerance) were positively related to OSP but not JSN [36]. This may be explained by some mediators like adipocytokines, which are involved in many metabolic processes in the body [37]. Besides, some animal studies also support our findings. Mooney et al. [38] and Iwata et al. [39] have demonstrated that high-fat diet increased the OSP diameter or volume in OA or type 2 diabetic mouse models. Similarly, Munter et al. [40] showed that the accumulation of low density lipoprotein within synovial lining cells led to increased activation of synovium and OSP formation. This interesting finding of the present study may give evidence to a better understanding of the pathogenesis of OA.

In addition to high prevalence of OA [18, 36, 41–44], Asian countries especially China, Japan and Korea are facing growing pressure of increasing prevalence of MetS, particular due to the dramatic changes in lifestyle in recent years [45, 46]. The study conducted by Gandhi et al. [47] showed that the prevalence of MetS in the Asian population was even higher than that in the white and black population. However, to the best of our knowledge, there was no large sample study yet examining the association between MetS and OA in the Chinese population. This is the first relatively large-scale study showing evidence that MetS diagnosed by the Chinese Diabetes Society criteria was associated with radiographic knee OA in the Chinese population. We speculate that the incidence and progression of MetS and OA are couple with each other including the severity of both diseases. This should be confirmed by further prospective cohort studies.

Among a variety of possible shared mechanisms between MetS and OA, chronic low-grade inflammation may be the most important one. An increasing number of researchers regarded MetS and OA as the low-grade inflammatory conditions, which were assessed by hsCRP levels sensitively [48]. However, the present cross-sectional study indicated that the multivariable adjusted association between MetS and knee OA remained unchanged after adding hsCRP into the multivariable model. This suggests that chronic low-grade inflammation may not be a very important mediator between MetS and OA. However, hsCRP is not the only marker of low-grade inflammation state, and other biomarkers of it should be examined in further studies exploring the association between MetS and OA. Interestingly, knee OA and MetS were associated regardless of BMI according to the NCEP-ATPIII (Asian revised version) criteria in the present study. The result is consist with a previous prospective cohort study [49] which showed that MetS was associated with increased risk of severe knee OA, independent of BMI. However, several previous studies [35, 50] also showed that the association between MetS and OA may mainly mediated by BMI. The disparities exist in these studies may be explained by the difference among population involved, and further cohort studies are needed to confirm it in the Chinese population.

There are still some limitations to this study. The cross-sectional design precludes causal correlations, so further prospective studies and intervention trials should be undertaken to establish a causal association between MetS and knee OA. Besides, pain was not taken into account in the present study and, therefore, the association between MetS and symptomatic knee OA cannot be examined. The present study also has several strengths. This is the first relatively large-scale study examined the association between MetS and OA in the Chinese population, and the first suggesting that MetS as a whole was associated with OSP, but not JSN. Meanwhile, several MetS definitions, including CDS criteria and NCEP-ATPIII (Asian revised version) criteria were used to assess MetS. In addition, the multivariable model was adjusted by a considerable number of potential confounding factors, especially hsCRP, which greatly improved the reliability of the results.

## Conclusions

The findings of the present study indicated that there was a positive association between the prevalence of MetS and knee OA. However, MetS as a whole was associated with the higher prevalence of knee OSP, but not JSN, which should shed light on our understanding the association between MetS and OA.

## Abbreviations

BMI: Body mass index
BP: Blood pressure
CDS: Chinese Diabetes Society
FPG: Fasting plasma glucose
hsCRP: High sensitivity C reactive protein
JSN: Joint space narrowing
K-L: Kellgren-Lawrence
MetS: Metabolic syndrome
NCEP-ATPIII: National Cholesterol Education Program Adult Treatment Panel III
OA: Osteoarthritis
OARSI: Osteoarthritis Research Society International
OR: Odds ratio
OSP: Osteophyte

## References

[1] Glyn-Jones S, Palmer AJ, Agricola R, Price AJ, Vincent TL, Weinans H, Carr AJ (2015). Osteoarthritis. Lancet.

[2] Zhuo Q, Yang W, Chen J, Wang Y (2012). Metabolic syndrome meets osteoarthritis. Nat Rev Rheumatol.

[3] Hart DJ, Doyle DV, Spector TD (1995). Association between metabolic factors and knee osteoarthritis in women: the Chingford study. J Rheumatol.

[4] Sturmer T, Brenner H, Brenner RE, Gunther KP (2001). Non-insulin dependent diabetes mellitus (NIDDM) and patterns of osteoarthritis. The Ulm osteoarthritis study. Scand J Rheumatol.

[5] Conaghan PG, Vanharanta H, Dieppe PAI (2005). Progressive osteoarthritis an atheromatous vascular disease?. Ann Rheum Dis.

[6] Dieppe PA, Lohmander LS (2005). Pathogenesis and management of pain in osteoarthritis. Lancet.

[7] Sellam J, Berenbaum FI (2013). Osteoarthritis a metabolic disease?. Joint Bone Spine.

[8] Katz JD, Agrawal S, Velasquez M (2010). Getting to the heart of the matter: osteoarthritis takes its place as part of the metabolic syndrome. Curr Opin Rheumatol.

[9] Velasquez MT, Katz JD (2010). Osteoarthritis: another component of metabolic syndrome?. Metab Syndr Relat Disord.

[10] Yamada T, Kawano H, Koshizuka Y, Fukuda T, Yoshimura K, Kamekura S, Saito T, Ikeda T, Kawasaki Y, Azuma Y (2006). Carminerin contributes to chondrocyte calcification during endochondral ossification. Nat Med.

[11] Kamekura S, Kawasaki Y, Hoshi K, Shimoaka T, Chikuda H, Maruyama Z, Komori T, Sato S, Takeda S, Karsenty G (2006). Contribution of runt-related transcription factor 2 to the pathogenesis of osteoarthritis in mice after induction of knee joint instability. Arthritis Rheum.

[12] Jones G, Ding C, Scott F, Glisson M, Cicuttini F (2004). Early radiographic osteoarthritis is associated with substantial changes in cartilage volume and tibial bone surface area in both males and females. Osteoarthr Cartil.

[13] Peng X (2011). China's demographic history and future challenges. Science.

[14] Gu D, Reynolds K, Wu X, Chen J, Duan X, Reynolds RF, Whelton PK, He J (2005). Prevalence of the metabolic syndrome and overweight among adults in China. Lancet.

[15] He Y, Jiang B, Wang J, Feng K, Chang Q, Fan L, Li X, Hu FB (2006). Prevalence of the metabolic syndrome and its relation to cardiovascular disease in an elderly Chinese population. J Am Coll Cardiol.

[16] Xi B, He D, Hu Y, Zhou D (2013). Prevalence of metabolic syndrome and its influencing factors among the Chinese adults: the China health and nutrition survey in 2009. Prev Med.

[17] Zhang Y, Xu L, Nevitt MC, Aliabadi P, Yu W, Qin M, Lui LY, Felson DT (2001). Comparison of the prevalence of knee osteoarthritis between the elderly Chinese population in Beijing and whites in the United States: the Beijing osteoarthritis study. Arthritis Rheum.

[18] Kang X, Fransen M, Zhang Y, Li H, Ke Y, Lu M, Su S, Song X, Guo Y, Chen J (2009). The high prevalence of knee osteoarthritis in a rural Chinese population: the Wuchuan osteoarthritis study. Arthritis Rheum.

[19] Li H, George DM, Jaarsma RL, Mao X (2016). Metabolic syndrome and components exacerbate osteoarthritis symptoms of pain, depression and reduced knee function. Ann Transl Med.

[20] Zeng C, Wei J, Li H, Yang T, Zhang FJ, Pan D, Xiao YB, Yang TB, Lei GH (2015). Relationship between serum magnesium concentration and radiographic knee osteoarthritis. J Rheumatol.

[21] Zeng C, Li H, Wei J, Yang T, Deng ZH, Yang Y, Zhang Y, Yang TB, Lei GH (2015). Association between dietary magnesium intake and radiographic knee osteoarthritis. PLoS One.

[22] Zhang Y, Zeng C, Li H, Yang T, Deng ZH, Yang Y, Ding X, Xie DX, Wang YL, Lei GH (2015). Relationship between cigarette smoking and radiographic knee osteoarthritis in Chinese population: a cross-sectional study. Rheumatol Int.

[23] Li H, Zeng C, Wei J, Yang T, Gao SG, Li YS, Luo W, Xiao WF, Xiong YL, Lei GH (2016). Relationship between soy milk intake and radiographic knee joint space narrowing and osteophytes. Rheumatol Int.

[24] Ding X, Zeng C, Wei J, Li H, Yang T, Zhang Y, Xiong YL, Gao SG, Li YS, Lei GH (2016). The associations of serum uric acid level and hyperuricemia with knee osteoarthritis. Rheumatol Int.

[25] Yang T, Ding X, Wang YL, Zeng C, Wei J, Li H, Xiong YL, Gao SG, Li YS, Lei GH (2016). Association between high-sensitivity C-reactive protein and hyperuricemia. Rheumatol Int.

[26] JH K, JS L (1957). Radiological assessment of osteo-arthrosis. Ann Rheum Dis.

[27] Altman RD, Gold GE. Atlas of individual radiographic features in osteoarthritis, revised. Osteoarthritis Cartilage. 2007;15(Suppl AA1–56) 10.1016/j.joca.2006.11.009 17320422

[28] Expert Panel on Metabolic Syndrome of Chinese Diabetes Society (2004). Recommendations on metabolic syndrome of Chinese diabetes society (Chinese). Chin. J Diabetes.

[29] Pang C, Jia L, Hou X, Gao X, Liu W, Bao Y, Jia W (2014). The significance of screening for microvascular diseases in Chinese community-based subjects with various metabolic abnormalities. PLoS One.

[30] Zhou H, Guo ZR, LG Y, XS H, BH X, Liu HB, Wu M, Zhou ZY (2010). Evidence on the applicability of the ATPIII, IDF and CDS metabolic syndrome diagnostic criteria to identify CVD and T2DM in the Chinese population from a 6.3-year cohort study in mid-eastern China. Diabetes Res Clin Pract.

[31] Tan CE, Ma S, Wai D, Chew SK, Tai ES (2004). Can we apply the National Cholesterol Education Program Adult Treatment Panel definition of the metabolic syndrome to Asians?. Diabetes Care.

[32] Liu J, Grundy SM, Wang W, Smith SJ, Vega GL, Wu Z, Zeng Z, Wang W, Zhao D (2006). Ethnic-specific criteria for the metabolic syndrome: evidence from China. Diabetes Care.

[33] Engstrom G, Gerhardsson DVM, Rollof J, Nilsson PM, Lohmander LS (2009). C-Reactive protein, metabolic syndrome and incidence of severe hip and knee osteoarthritis. A population-based cohort study. Osteoarthr Cartil.

[34] Han CD, Yang IH, Lee WS, Park YJ, Park KK. Correlation between metabolic syndrome and knee osteoarthritis: data from the Korean National Health and nutrition examination survey (KNHANES). BMC Public Health. 2013:13603. 10.1186/1471-2458-13-603 PMC3691727 23800128

[35] Visser AW, de Mutsert R, le Cessie S, den Heijer M, Rosendaal FR, Kloppenburg M (2015). The relative contribution of mechanical stress and systemic processes in different types of osteoarthritis: the NEO study. Ann Rheum Dis.

[36] Yoshimura N, Muraki S, Oka H, Kawaguchi H, Nakamura K, Akune T (2011). Association of knee osteoarthritis with the accumulation of metabolic risk factors such as overweight, hypertension, dyslipidemia, and impaired glucose tolerance in Japanese men and women: the ROAD study. J Rheumatol.

[37] Richter M, Trzeciak T, Owecki M, Pucher A, Kaczmarczyk J (2015). The role of adipocytokines in the pathogenesis of knee joint osteoarthritis. Int Orthop.

[38] Mooney RA, Sampson ER, Lerea J, Rosier RN, Zuscik MJ (2011). High-fat diet accelerates progression of osteoarthritis after meniscal/ligamentous injury. Arthritis Res Ther.

[39] Iwata M, Ochi H, Hara Y, Tagawa M, Koga D, Okawa A, Asou Y (2013). Initial responses of articular tissues in a murine high-fat diet-induced osteoarthritis model: pivotal role of the IPFP as a cytokine fountain. PLoS One.

[40] de Munter W, Blom AB, Helsen MM, Walgreen B, van der Kraan PM, Joosten LA, van den Berg WB, van Lent PL (2013). Cholesterol accumulation caused by low density lipoprotein receptor deficiency or a cholesterol-rich diet results in ectopic bone formation during experimental osteoarthritis. Arthritis Res Ther.

[41] Ho-Pham LT, Lai TQ, Mai LD, Doan MC, Pham HN, Nguyen TV (2014). Prevalence of radiographic osteoarthritis of the knee and its relationship to self-reported pain. PLoS One.

[42] Salve H, Gupta V, Palanivel C, Yadav K, Singh B (2010). Prevalence of knee osteoarthritis amongst perimenopausal women in an urban resettlement colony in South Delhi. Indian J Public Health.

[43] Yoshimura N, Muraki S, Oka H, Mabuchi A, En-Yo Y, Yoshida M, Saika A, Yoshida H, Suzuki T, Yamamoto S (2009). Prevalence of knee osteoarthritis, lumbar spondylosis, and osteoporosis in Japanese men and women: the research on osteoarthritis/osteoporosis against disability study. J Bone Miner Metab.

[44] Zeng QY, Zang CH, Li XF, Dong HY, Zhang AL, Lin L (2006). Associated risk factors of knee osteoarthritis: a population survey in Taiyuan, China. Chin Med J.

[45] Nestel P, Lyu R, Low LP, Sheu WH, Nitiyanant W, Saito I, Tan CE (2007). Metabolic syndrome: recent prevalence in east and southeast Asian populations. Asia Pac J Clin Nutr.

[46] Hoang KC, Le TV, Wong ND (2007). The metabolic syndrome in east Asians. J Cardiometab Syndr.

[47] Gandhi R, Razak F, Tso P, Davey JR, Mahomed NN (2010). Asian ethnicity and the prevalence of metabolic syndrome in the osteoarthritic total knee arthroplasty population. J Arthroplast.

[48] Huffman KM, Kraus WE (2012). Osteoarthritis and the metabolic syndrome: more evidence that the etiology of OA is different in men and women. Osteoarthr Cartil.

[49] Monira HS, Wang Y, Cicuttini FM, Simpson JA, Giles GG, Graves S, Wluka AE (2014). Incidence of total knee and hip replacement for osteoarthritis in relation to the metabolic syndrome and its components: a prospective cohort study. Semin Arthritis Rheum.

[50] Puenpatom RA, Victor TW (2009). Increased prevalence of metabolic syndrome in individuals with osteoarthritis: an analysis of NHANES III data. Postgrad Med.

